# Therapeutic Fasting as a Novel Approach to Mitigate Musculoskeletal Symptoms in Breast Cancer Patients undergoing Aromatase Inhibitor Therapy: A Feasibility Study Protocol

**DOI:** 10.1177/15347354261426272

**Published:** 2026-03-10

**Authors:** Henriette Meyer, Daniela A. Koppold, Osaid Shaker, Laura Eden, Lisa Schiffmann, Stefan Konigorski, Holger Cramer, Kyung-Eun Anna Choi, Hermann Einsele, Marcela Winkler, Claudia Löffler

**Affiliations:** 1 Department of Internal Medicine II, University Hospital of Würzburg, Germany; 2 Institute of Social Medicine, Epidemiology anf Health Economics, corporate member of Freie niversität Berlin and Humboldt-Universität, Charité – Universitätsmedizin Berlin, Germany; 3 Department of Internal Medicine and Nature-based Therapies, Immanuel Hospital Berlin, Germany; 4 Department of Pediatrics, Division of Oncology an Hematology, Charité - Universitätsmedizin Berlin, Germany; 5 Technische Universität Dresden, Germany; 6 Robert Bosch Center for Integrative Medicine and Health, Bosch Health Campus, Stuttgart, Germany; 7 Digital Health-Machine Learning Group, Digital Health Center, Hasso Plattner Institute, University of Potsdam, Germany; 8 Insititute of General Practice and Interprofessional Care, University Hospital Tübingen, Germany; 9 Brandenburg Medical School Theodor Fontane, Centre for Health Services Research, Neuruppin, Germany; 10 Health Services Research Group, Faculty of Medicine/Dentistry, Danube Private University, Krems, Austria; 11 Evidence Based Practice in Brandenburg – A JBI Affiliated Group, Germany; 12 Robert-Bosch-Hospital, Stuttgart, Germany

**Keywords:** breast cancer, prolonged fasting, therapeutic fasting, caloric restriction, AIMSS/AIA, nutrition, musculoskeletal pain, aromatase inhibitors

## Abstract

**Introduction:**

Aromatase inhibitor-induced musculoskeletal symptoms (AIMSS) or aromatase inhibitor-induced arthralgia (AIA), common side effects of long-term adjuvant endocrine therapy for breast cancer patients, can significantly affect quality of life. Preliminary clinical evidence suggests that fasting may alleviate these symptoms, as demonstrated in conditions such as rheumatoid arthritis and fibromyalgia. This study aims to evaluate the feasibility and acceptability of prolonged therapeutic fasting program, including structured behavioral and educational support, in patients with AIMSS/AIA and explore the applicability of validated measurement tools for assessing symptom burden in preparation for a confirmatory bicentric trial.

**Methods:**

This is a bicentric, single-arm, prospective pilot study. We will enroll 54 participants undergoing endocrine therapy with aromatase inhibitors who suffer from AIMSS/AIA. All participants will receive a 7-day online- prolonged therapeutic fasting intervention (max. 350 kcal/day), consisting of vegetable juices and broths under medical supervision. The primary outcome is the feasibility of prolonged therapeutic fasting during aromatase inhibitor therapy assessed through participant adherence to the fasting protocol and completion rate. As a secondary endpoint, we will assess the feasibility of using validated measurement instruments to collect data on symptoms, quality of life, mindfulness, stress, and sleep quality in preparation for a confirmatory trial. The following well established tools will be used: Numeric Rating Scale (NRS), Visual Analog Scale (VAS), Brief Pain Inventory (BPI), Fibromyalgia Impact Questionnaire (FIQ), Health Assessment Questionnaire–Disability Index (HAQ-DI), WHO-5 Well-Being Index (WHO-5), Mindful Attention Awareness Scale (MAAS), Brief Fatigue Inventory (BFI) and Pittsburgh Sleep Quality Index (PSQI). Specifically, we aim to explore their applicability by evaluating completion rates, participant acceptability, and data completeness within this patient population. Exploratory parameters, including changes in dietary habits, anthropometric data, and cancer-related fatigue will be tracked to contextualize the feasibility findings and inform outcome selection for a future confirmatory trial. An accompanying N-of-1 trial using a mobile application (StudyU) and qualitative interviews will give more insight into individual and subjective changes.

**Conclusion:**

If the intervention proves feasible and well accepted, these findings will support the development of a confirmatory trial designed to investigate the potential clinical benefits and underlying mechanisms of fasting in AIMSS/AIA. Long-term studies incorporating biomarkers and microbiota analysis could further explore the mechanisms underlying symptom improvement and guide personalized treatment strategies.

Trial registration: ClinicalTrials.gov.

NCT06172088. Registered 14 December 2023.

URL: https://clinicaltrials.gov/study/NCT06172088.

## Introduction

Breast cancer is the most frequently diagnosed cancer and the leading cause of death among women worldwide.^1^ Advances in early detection, diagnostic precision and therapeutic approaches have significantly improved survival rates, leading to an increasing number of women undergoing long-term adjuvant therapies.^2^ Among these, aromatase inhibitors (AIs) are the standard therapy for postmenopausal women with early-stage, hormone receptor-positive breast cancer, which accounts for over 80% of all breast cancer cases.^3,4^ Typically prescribed for up to 10 years, AIs significantly reduce recurrence risk and improve survival outcomes.^5,6^ However, despite these therapeutic benefits, their use is frequently associated with adverse effects, particularly aromatase inhibitor-induced arthralgia (AIA) and aromatase inhibitor-induced musculoskeletal symptoms (AIMSS), affecting up to 74% of treated patients.^7,8^ These symptoms often lead to an increase in psychosomatic distress and physical disability, followed by poor adherence and therapy discontinuation, which results in higher rates of breast cancer recurrence and all-cause mortality.^9^

AIMSS/AIA often presents as arthralgia, osteopenia and an increased risk of fractures.^10-12^ These manifestations are largely attributed to the hypoestrogenic state induced by AIs, which elevates pro-inflammatory cytokines and interleukins, stimulating bone resorption and impairing bone formation.^13,14^ Understanding the pathophysiological mechanisms of AIMSS/AIA is essential for developing effective therapeutic strategies; however, these mechanisms remain only partially understood, as estrogen deficiency not only affects bone health but also influences joint pain, inflammation, and immune function. Estrogen normally exerts chondroprotective and antinociceptive effects, and its absence results in increased pain perception and autoimmune dysregulation.^8,15,16^ Radiological evidence further supports these findings, showing increased fluid in joints and tendon sheaths, which contributes to pain and inflammation.^17-19^

Many chronic diseases are aggravated by diet induced inflammation. Caloric restriction (CR) has been shown to reduce inflammation and oxidative damage while enhancing insulin sensitivity and energy metabolism.^20-23^ Most studies report beneficial effects with a moderate caloric restriction of about 20% to 40% of daily energy intake.^24^ In contrast, therapeutic fasting regimens—such as the one applied in our study—induce a short-term, profound energy reduction of approximately 80% to 85% (≈350 kcal per day), consistent with the Buchinger fasting method.^25,26^ Additionally, CR shapes the gut microbiota, promoting anti-inflammatory short-chain fatty acids (SCFA),^27,28^ improving gut barrier integrity and maintaining intestinal homeostasis.^29^ While no previous studies have directly addressed therapeutic fasting in the context of AIMSS/AIA, related work in inflammatory musculoskeletal conditions (eg, rheumatoid arthritis) has shown promising results that could also be relevant for the discussed triggering mechanisms of AIMSS/AIA. Evidence from these related conditions provides an important clinical parallel supporting the underlying biological rationale. For instance, clinical studies in rheumatoid arthritis and fibromyalgia demonstrated reductions in pain, stiffness, and inflammatory markers following fasting interventions, suggesting beneficial effects on both immune regulation and musculoskeletal function.^25,26,30^ These findings support the rationale for evaluating fasting as a potential strategy to mitigate AIMSS/AIA symptoms. Recent studies have for example, demonstrated that a modulation of intestinal microbiota composition through therapeutic fasting could reduce arthritogenic and pro-inflammatory species, decreasing systemic markers such as IL-6 and zonulin, and enhancing metabolic flexibility.^31^ These microbial shifts may synergize with fasting-induced increases in short-chain fatty acids like butyrate, known to promote gut barrier function and induce regulatory T cells.^32^ In parallel, fasting increases circulating ketone bodies such as β-hydroxybutyrate, which cross the blood–brain barrier and upregulate brain-derived neurotrophic factor (BDNF), contributing to neuroplasticity and potentially modulating pain perception.^33,34^ Fasting has also been shown to stimulate endogenous stress pathways—including cortisol, catecholamines, and hypothalamic orexin A signaling—which may enhance mood and activate intrinsic analgesic systems.^35^

Such mechanisms emphasize its potential to modulate inflammation and support health.

In recent years therapeutic fasting has garnered attention for its potential to reduce inflammation, enhance treatment efficacy, and alleviate side effects in various medical conditions, including metabolic disorders, cardiovascular diseases, and cancer.^24,36-40^ Its simplicity and low-risk profile make fasting an accessible therapy that encourages patients to take an active role in managing their health.^39^

Previous work by our group in Berlin has demonstrated pain-reducing effects of a 7 to 10-day therapeutic fasting intervention providing approximately 200 to 500 kcal/day according to the Buchinger therapeutic fasting on fibromyalgia-like symptoms.^25,26^ More recently, an exploratory randomized controlled trial on fasting and plant-based nutrition in rheumatoid arthritis showed favorable effects on disease activity following a 7-day fasting period.^30^ In line with these studies, the present trial applies a 7-day, calorie-restricted therapeutic fasting program (~350 kcal/day) following the Buchinger method, including structured preparation and refeeding phases under medical supervision. Despite these findings, therapeutic fasting has not yet been investigated in the context of AIMSS/AIA. Previous studies in rheumatoid arthritis have shown clinically relevant improvements during the fasting period itself. Observational data from Buchinger fasting programs also report improvements between the beginning and end of the fasting period.^41^ These findings support the assumption that relevant short-term changes can occur during fasting.^42,43^ Based on these considerations, the research question arises as to whether prolonged therapeutic fasting can reduce physical limitations due to AIMSS/AIA and improve quality of life.

Various fasting regimens have been described in the literature, including intermittent fasting, time-restricted feeding, alternate-day fasting, fasting-mimicking diets, and therapeutic fasting.^44^ These approaches differ in duration, caloric intake, and level of medical supervision. Among them, the Buchinger therapeutic fasting method represents one of the best-established clinical models in Europe. It provides a short-term but profound caloric reduction of approximately 80% to 85% (≈350 kcal/day) for 7 days under medical supervision, combined with preparatory and refeeding phases. This regimen was selected for the present study because previous clinical research has reported anti-inflammatory, metabolic, and pain-relieving effects in patients with comparable chronic inflammatory and musculoskeletal conditions.^26,30,40^ These findings provided the rationale and motivation to explore the feasibility of this fasting approach in the context of AIMSS/AIA.

In addition, plant-based and high-fiber diets have been shown to complement fasting by reducing systemic inflammation, improving metabolic flexibility, and supporting gut microbiota composition. These dietary principles are increasingly integrated into fasting-based interventions to sustain long-term benefits after the fasting phase.^45,46^

Adherence is a central challenge in fasting and other lifestyle-based interventions. Structured supervision, regular medical contact, and group sessions have been shown to enhance safety, motivation, and adherence in fasting programs by providing education, social support, and monitoring.^41,47,48^

We have designed this exploratory pilot study to determine the feasibility and acceptability of implementing a prolonged therapeutic fasting program, including structured behavioral and educational support, intervention in breast cancer patients with AIMSS/AIA, and to explore the applicability of validated measurement tools for future use. An accompanying N-of-1 trial using a mobile application (StudyU) and qualitative interviews in the form of a guided focus group formed by up to 12 participants, will provide more insight into individual and subjective changes during the study period.

## Materials and Methods

### Study Setting

This is a bicentric, single-arm, prospective pilot study designed to evaluate the feasibility of prolonged therapeutic fasting in patients with AIMSS/AIA. To ensure methodological transparency and reproducibility, the study reporting follows the CONSORT extension for pilot and feasibility studies reporting guidelines (Eldridge et al., 2016), and the study protocol adheres to these standards. The study protocol was approved by the Ethics Committees of the Bavarian State Chamber of Physicians (Ethik-Kommission der Bayerischen Landesärztekammer, reference 35/23-me, approval date 22 June 2023) and the State Chamber of Physicians of Baden-Württemberg (Ethik-Kommission der Landesärztekammer Baden-Württemberg, reference B-F-2023-069, approval date 10 July 2023). The study will be conducted in accordance with the Declaration of Helsinki. The patient handout, which describes the course of the intervention in detail, the standard operating procedure for adapting medication, the declaration of consent and a questionnaire created for the study to record eating habits, exercise behavior and fasting experiences, are available in Supplemental Files 1-4. The study was registered at ClinicalTrials.gov NCT06172088, registration date 14 December 2023), shortly after the start of recruitment, which began in November 2023

All participants who meet the inclusion criteria (see Table 1) and provide informed consent will be enrolled. The recruitment period is scheduled to last a total of 18 months.

**Table 1.** Inclusion and Exclusion Criteria.

Inclusion criteria	Exclusion criteria
• Patients with curable hormone receptor-positive breast cancer who are receiving adjuvant treatment with aromatase inhibitors for at least 3 mo, and experiencing significant joint pain (NRS >4) • 18 y or older • Written consent for the storage and sharing of personal medical data in accordance with the study protocol	• Any medical condition that contraindicates fasting, especially: ○ Long-term medication unrelated to endocrine therapy (e.g. Marcumar, Lithium, antiepileptic drugs) ○ Mental condition preventing the provision of legally valid consent ○ Eating disorder or BMI <20 kg/m^2a^ ○ Uncontrolled cerebral seizures ○ Type 1 or type 2 diabetes mellitus ○ Severe oncological comorbidity or ongoing chemotherapy ○ Pregnancy or breastfeeding
	• Inability to speak or understand German • Participation in another fasting study

a Fasting interventions are not recommended in underweight populations according to current clinical nutrition guidelines Muscaritoli et al.^49^; For patients over the age of 65, a BMI < 20 kg/m^2^ might be a risk in fasting interventions.^50^

#### Participant Timeline

**Figure 1.**
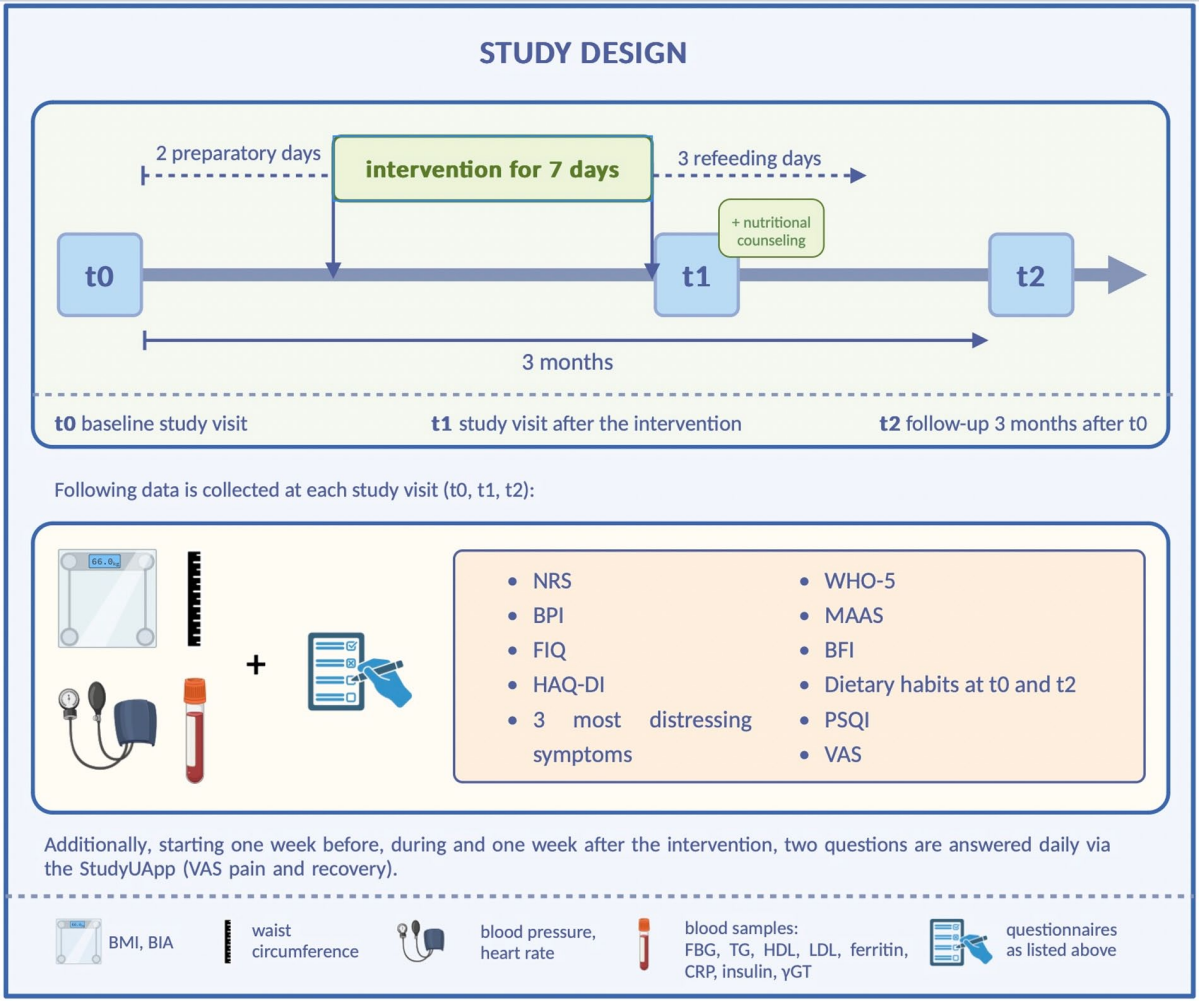
Measurement times of the FREE-AI pilot study. Unless explicitly stated, all measurements are carried out at all 3 time points. Source: This image was created with BioRender.com. Abbreviations: BFI, brief fatigue inventory; BIA, bioelectrical impedance analysis; BMI, body-mass index; BPI, brief pain inventory; CRP, C-reactive protein; FBG, fasting plasma glucose; FIQ, fibromyalgia impact questionnaire; gGT, gamma-glutamyl transferase; HAQ.DI, health assessment questionnaire-disability index; HDL/LDL, high/low-density lipoprotein cholesterol; MAAS, mindful attention awareness scale; NRS, numeric rating scale; PSQI, Pittsburgh sleep quality index; TG, triglycerides; VAS, visual analog scale; WHO-5, World Health Organization’s five-point index.

The first participants were recruited in November 2023. It is planned to enroll 54 participants who will visit their respective study centers for 3 study visits: at baseline (*t*0), after completion of the 12-day fasting program, including 2 preparation days, 7 fasting days and 3 refeeding days (*t*1), (*t*1), and after 3 months (*t*2; see Figure 1 for illustration). The duration per patient is 12 weeks. Following the screening at baseline *t*0, all patients receive the intervention (prolonged therapeutic fasting program including structured behavioral and educational support).

### Recruitment

Patients are being recruited in 2 different specialized centers for integrative Medicine and Oncology in Germany: the Department of Integrative Medicine at the Robert Bosch Hospital, Stuttgart, and the Department of Internal Medicine II—Oncology at the University Hospital Würzburg. Participants will be recruited through informational materials (flyers, posters, and digital announcements) distributed in the participating hospitals and cooperating departments, as well as through internal communication channels and staff meetings within the involved institutions. Information about the study was also shared at departmental briefings and relevant scientific events. The recruitment procedure was reviewed and approved by both responsible ethics committees, and participation is entirely voluntary following written informed consent.

During the first screening, which is held prior to the first study visit (*t*0), eligibility is determined by the investigators and only if all inclusion and no exclusion criteria (see Table 1) apply can the patient be included in the study.

Patients also receive detailed information on both the background and protocol of the study. Each patient will sign a consent form, agreeing to the collection and use of their data. The informed consent form can be found in Supplemental File 1.

### Eligibility Criteria

Participants will be recruited according to the following inclusion and exclusion criteria, which were defined to ensure patient safety and the feasibility of the fasting intervention.

### Interventions

The study intervention is a 7-day online accompanied prolonged therapeutic fasting program including structured behavioral and educational support with a maximum caloric intake of 350 kcal per day. To ensure participant safety and tolerability, the intervention includes a structured preparatory phase with dietary adjustments, such as light vegetarian meals prior to fasting, and a gradual refeeding period afterward. These phases are intended to minimize gastrointestinal distress and abrupt metabolic changes. Participants are provided with precise instructions for juice and broth preparation and quantities. While the dietary protocol is standardized, adjustments within the permitted range are encouraged based on individual tolerance. Nutritional support is reinforced through frequent contact with the study team and 24-hour telephone assistance. Adherence and safety are monitored clinically, and predefined withdrawal criteria are in place. The modified fasting protocol has been applied in previous studies and shown to be well tolerated.^26^ Online meetings include two 90 minute meetings, 4 sessions of 60 minutes each and, if required, one additional 30-minute meeting. These meetings serve to provide medical monitoring, fasting-related education, guidance on refeeding, and psychosocial support to ensure participant safety and adherence. Online meetings include two 90-minute meetings, 4 sessions of 60 minutes each and, if required, one additional 30-minute meeting. These meetings serve to provide medical monitoring, fasting-related education, guidance on refeeding, and psychosocial support to ensure participant safety and adherence. The fasting program ensures close supervision and support by both a specifically trained nutritionist and a physician. Adherence to the fasting regimen is defined as completing at least 5 out of 7 fasting days within these parameters. All nutritionists hold a diploma from one of the certified fasting academies of Germany.^51,52^ Furthermore, all members of the study personnel are trained and certified. The fasting program ensures close supervision and support by both a specifically trained nutritionist and a physician.

Criteria for discontinuation are defined to ensure participant safety and to appropriately document feasibility. Although prolonged fasting is generally considered safe in supervised settings, participants may discontinue the fasting intervention at any time in case of intolerance. If a participant consumes more than the permitted caloric intake on a given day, this deviation will be documented as part of the feasibility assessment, but the participant will not be withdrawn from the study for this reason alone.

Adverse events (AEs) and severe adverse events (SAEs) are continuously documented according to the Common Terminology Criteria for Adverse events (CTCAE), Version 5.0 (U.S. Department of Health and Human Services, 2017). Should relevant (S)AEs occur—such as marked dizziness, clinically concerning weight loss (eg, BMI dropping below 20 kg/m^2^), or other symptoms that raise safety concerns—the study physician will evaluate the situation and decide whether continuation of the fasting protocol is appropriate. In cases of doubt, the fasting intervention will be discontinued as a precaution. Participants will, however, remain enrolled in the study unless safety considerations explicitly require study removal. All discontinuations of the fasting intervention and the underlying reasons will be recorded in detail.

Personal doubts regarding the ability to adhere to the intervention are a major factor influencing compliance. Although the adherence cannot be comprehensively monitored in the outpatient setting, close supervision and support will be provided. The online group meetings can help build a sense of community, potentially affecting compliance in a positive way.

Ongoing medical treatments or changes to them will be documented on each study visit. For some medications (eg, diuretics, antihypertensives, oral antidiabetics and others) a dose adjustment may be necessary, as fasting has effects on metabolism and therefore various biochemical parameters. For more information on possible and necessary changes in medication, please refer to Supplemental File 2.

The fasting method as used in our intervention is based on the protocol established by the Charité University Outpatient Clinic for Naturopathy, Immanuel Hospital Berlin, and has already been evaluated in various clinical studies.^25,30,40^

Participants are only allowed to consume vegetable juices (without sugar) and vegetable broths prepared at home according to standardized recipes provided by the study team to ensure a daily intake of ≤350 kcal. The juices are made from fresh vegetables without added sugar. Participants may drink herbal teas (without caffeine or theine), and water ad libitum; however, tea should be consumed without milk, cream, or sweeteners. A total fluid intake of 2 to 3 l per day is recommended according to the fasting guidelines of the German Fasting Academy (Ärztegesellschaft Heilfasten & Ernährung e.V.) to maintain hydration and metabolic stability. The evening before the intervention begins, there is an online group meeting to create room for sharing experiences and concerns with each other and to address questions.

During the 2 pre-fasting days, participants gradually reduce general food intake and avoid stimulants such as nicotine, coffee, black or green tea. Only easily digestible, carbohydrate-containing foods such as rice, oats, and steamed vegetables are consumed to support the metabolic transition to fasting, maintain glycogen stores, and minimize withdrawal symptoms. This preparatory phase follows the Buchinger fasting guidelines.^40,51^ Following the preparatory days, the participants will begin the actual fasting for a further 7 days, with vegetable juice (150 ml in the morning and 150 ml at lunchtime) and vegetable broth (250 ml in the evening). On the final day, the group gathers again online to break the fast together. In the following 3 days, food intake is slowly increased. All participants receive a study-specific flyer with information as well as recipe suggestions for both the preparatory, fasting and refeeding days. For more details regarding the fasting intervention, please refer to Supplemental File 3.

During the fasting week, sufficient outdoor activity (approx. 1 hour/day) in the form of gymnastics, cycling, swimming or walking is recommended. Mindfulness and awareness exercises are incorporated in the online meetings and we encourage incorporating periods of rest, including autogenic training, light yoga, meditation or Qi Gong practice.

To enhance understanding and motivation to follow a plant-based, high-fiber diet and to integrate intermittent fasting or time restricted eating (16/8 hours) after completion of the fasting intervention, participants receive an additional online nutritional counseling (90 minutes) at *t*1. The 16/8 approach is not part of the fasting program itself but serves as a post-intervention strategy to promote long-term healthy eating patterns.

At the end of treatment visits participants have the opportunity to discuss the results of their blood tests and anthropometric characteristics together.

### Outcomes

#### Primary Outcome

The primary outcome of this study is to test the feasibility of the recruitment and intervention.

The intervention comprises a prolonged therapeutic fasting program combined with structured behavioral and educational support (medical supervision, group meetings, and online guidance).

Feasibility of recruitment will be evaluated by:

- The number of patients screened and included within the 18-month recruitment period,
- The proportion of eligible patients who consent to participate, and
- The time required to enroll a sufficient number of participants to start a group.

Feasibility of the intervention will be assessed by:

- Treatment-related retention and dropout rates,
- Reasons for dropouts,
- Adherence to the fasting protocol on both the participants’ and therapists’ side.
- Adherence is monitored via the StudyU app, which records daily fasting compliance. Adherence is defined as completing at least 5 out of 7 fasting days while maintaining a caloric intake ≤350 kcal/day. If more than 30% of participants fail to meet this criterion, the intervention will be considered not feasible.

The StudyU app—an app to design and perform N-of-1 trials—is used to assess adherence daily. Adherence is defined as completing at least 5 out of 7 fasting days while maintaining a caloric intake of ≤350 kcal/day. If more than 30% of participants fail to meet this criterion, the intervention will be considered not feasible.

Feasibility of the assessments will be determined by:

- The proportion of planned assessments that are completed
- The duration of assessment visits, and
- The number of dropouts due to assessment burden).

A completion rate of at least 75% is required to determine feasibility.

Acceptability of the intervention will be explored through:

- Qualitative data from guided focus groups, and
- Dropout reasons related to fasting burden.

Applicability of the measurement tools will be evaluated through completion rates, participant feedback, and data completeness for the validated questionnaires (NRS, VAS, BPI, FIQ, HAQ-DI, WHO-5, MAAS, BFI and PSQI).

Exploratory outcome for future trials:

With regard to a confirmatory study, an additional research question concerns the identification of a potential primary outcome parameter which validated questionnaire may best capture AIA/AIMSS symptom burden.

#### Secondary and Exploratory Outcomes

Four validated measurement instruments will be used to assess symptom burden at each study visit, see Figure 1 for illustration. Both the Numeric Rating Scale (NRS) and Visual Analog Scale (VAS) are widely recognized and validated measures of pain intensity. The Brief Pain Inventory (BPI) is used to assess the severity and location of pain, the effects of current medication or treatment, and its impact on mood.^52^ The Fibromyalgia Impact Questionnaire (FIQ) is a brief, self-administered, 10-item instrument designed to evaluate multiple dimensions of health status, including physical functioning, work status, depression, anxiety, sleep, pain, stiffness, fatigue, and overall well-being.^53^ Lastly, the Health Assessment Questionnaire-Disability Index (HAQ-DI) is a validated tool developed to measure the degree of difficulty adults with rheumatoid arthritis face in performing activities of daily life.^54^ Patients are also asked to list the 3 most bothersome symptoms and rate their severity on a 0 to 10 scale.

Even if a feasibility study lacks the power to detect efficacy effects, we have decided to integrate a number of questionnaires into the study in order to obtain a general tendency regarding possible effects that should be investigated in a confirmatory study. These questionnaires will be analyzed descriptively in order to evaluate, in the sense of exploratory testing, whether effects could be expected on average. Therefore, the following questionnaires are included at each study visit:

The World Health Organization’s 5-point index (WHO-5) is used to assess mental well-being,^55^ while mindfulness and awareness are measured using the Mindful Attention Awareness Scale (MAAS).^56^ The Brief Fatigue Inventory (BFI) is designed to assess cancer-related fatigue^57,44^ and the Pittsburgh Sleep Quality Index (PSQI) is used to measure subjective sleep quality.^58^

#### Additional Parameters

In order to describe the patient collective as accurately as possible, the following additional parameters will be recorded. At baseline sociodemographic data and medical history will be documented. Before starting the fasting intervention, participants are asked to report on their dietary habits by using a standardized questionnaire, please refer to Supplemental File 4. Besides dietary habits and the intake of stimulants, personal experiences with fasting as well as physical activity behavior are also recorded. Possible changes in dietary habits initiated by the fasting and nutritional counseling are documented at *t*2 (Supplemental File 4).

At each study visit (*t*0, *t*1 and *t*2) anthropometric data and adverse events are documented, and blood samples are offered on an optional basis. Anthropometric data include weight, height, BMI, heart rate, blood pressure, waist circumference and Bioelectrical impedance analysis (BIA) measurement. BIA will be used to assess for example, fat-free mass (FFM), skeletal muscle mass (SMM), total body water (TBW), intracellular and extracellular water (ICW/ECW), and phase angle (PA). To ensure reliable measurements, all assessments follow our institutional SOP, which includes standardized conditions such as: no food intake for at least 3 to 4 hours before measurement, adequate hydration the day before, avoidance of alcohol and caffeine for 24 hours and intense physical activity for 12 hours, and voiding the bladder immediately beforehand. Because the first BIA assessment occurs before the start of fasting and the second after the 3 day recovery period, these requirements can be met. The optional blood tests are collected primarily to provide participants with individualized feedback on metabolic and nutritional parameters at the end of the intervention. In addition, together with the anthropometric assessments, they serve as an additional evaluation of participants’ nutritional and general health status to ensure appropriate medical supervision and counseling before and during the fasting intervention. Blood biomarkers include fasting plasma glucose (FBG), gamma-GT, triglycerides (TG), high- and low-density lipoprotein-cholesterol (HDL, LDL), ferritin, CRP and insulin. Fatty liver index (FLI) and HOMA Index are calculated. The specimens are not stored for any further use.

In order to gain a better understanding of the individual experiences of the participants during the fasting process, they were asked to provide daily feedback in an N-of-1 trial app. Two questions will be posed daily via the StudyU app (VAS [Visual Analog Scale]; pain and fatigue), starting 1 week before, during and until 1 week after the intervention. This helps to assess the individual symptom burden. To evaluate the accompanying N-of-1 trials using the StudyU app, we aim to use a mixed methods approach, combining qualitative analysis of individual experiences with descriptive analysis of log report data.

After having completed their fasting intervention, up to 12 people will be conveniently sampled from the study cohort for a guided focus group (1 online session, lasting approx. 2 1⁄2 hours) to explore general acceptance as well as beneficial and inhibiting factors for the implementation of prolonged fasting. The German version of the semi-structured focus group guide is provided in Appendix (FREE-AI_Focus group guidelines). The discussion will be audio-recorded, transcribed verbatim, pseudonymized, and analyzed using a deductive-inductive content analysis approach. The deductive process of analysis will be guided by a socio-ecological framework introduced by Hull et al^59^ A similar approach was previously adopted in a qualitative study exploring the feasibility of a time-restricted eating intervention.^60^ A structured coding approach will be applied (2 coders will independently code qualitative data and discuss discrepancies) to identify key themes and patterns related to participants’ experiences, barriers, and facilitators of fasting adherence. Qualitative data will be managed using MAXQDA Software (Köln, Germany). (Supplemental File 5: Focus group guidelines)

### Sample Size and Statistical Analysis

The primary objective of the present study is its feasibility, assessed qualitatively and quantitatively as described in the methods section. Response rates and study compliance, as well as treatment adherence and dropout rates, will be calculated as the proportion of patients to whom a characteristic will be applied (eg, participation in follow-up measure) from the total population considered (all included patients) and will be reported as a percentage with corresponding 95% confidence interval (95% CI). Reasons for individual days of absence or study dropout will be reported qualitatively and quantitatively.

Descriptive statistics will be used to report baseline characteristics, including sociodemographic data and to evaluate possible effects that should be investigated in a confirmatory study. Given that this is a feasibility study, no statistical hypothesis testing or regression models will be applied, as the study is not powered to detect statistically significant effects. In order to inform the planning of a future confirmatory trial, we will obtain intervention effect estimates that is, assess whether pain and fatigue scores differ between intervention and non-intervention periods, both at the individual level and aggregated across participants. These analyses are exploratory, will estimate immediate average effects of applying the fasting intervention and are based on the assumption that the effects of fasting can be seen already within a few days. To this aim, we will apply linear regression models and Bayesian linear regression models, each with the pain and fatigue outcome variable assessed daily through the StudyU app, and an intervention indicator variable (*T*0 vs *T*1 and vs *T*2, respectively) as predictor. From linear regression models, we will obtain parameter estimates of mean differences between *T*0 and *T*1, and *T*0 and *T*2, and their standard error estimates, and from the Bayesian regression models we will obtain estimates of the posterior distribution of the treatment effects. These models will be fitted for each participant separately, and also together across participants. Together, both will give information about the size and variation of intervention effects. As further exploratory analyses, we will include linear time trends in the models, and the results may allow to generate hypotheses on possible underlying mechanisms. We will test assumptions of parametric models such as normality of residuals.

As this is a feasibility study, no formal power calculation was required for the primary objective. The target sample size of 54 participants was determined based on practical feasibility considerations, including recruitment capacity and an anticipated dropout rate of 10%. The sample size was chosen with uncertainty regarding the expected effect size in the confirmatory study. Since one of the objectives of the pilot study was to determine the appropriate primary endpoint for the main study, it was not yet possible to determine the effect size for the main study, which meant that its required sample size could not be adequately determined. As a result, we had to resort to rules of thumb for the pilot study’s sample size. Depending on the approach, these rules of thump range from 20 to 70 patients for a 2-arm randomized pilot trial. Whitehead et al (2016) evaluated these rules mathematically and showed that, even under conservative assumptions and very small expected effect sizes in the subsequent main trial, a 2-arm pilot study with approximately 50 participants provides adequate precision at 80% power (or about 75 participants at 90% power). Given that our study follows a single-arm pilot design a target sample size of 54 patients (including anticipated dropouts) can be considered conservative. The assumed 10% dropout rate reflects expected fasting-related discontinuation observed in previous fasting studies, while higher overall dropout rates reported in other studies were largely attributable to ongoing chemotherapy rather than fasting-specific tolerability.^61^

### Allocation Not Applicable

#### Blinding

The study is an open-label study, as blinding of the participants is neither possible for single-arm studies nor in fasting interventions.

### Data Collection

The measures and cut offs for testing feasibility and also the assessment tools for the other research questions have been described above. The endpoints in the form of questionnaires or patient reported outcomes (PROs) are recorded via the StudyU app^62^ and electronic Case Report Form (eCFRs) via lime survey. The StudyU platform (https://studyu.health) allows intervention studies to be performed digitally while adhering to data protection regulations.

The StudyU applications are written in Flutter, an open-source framework. The source code is publicly available on GitHub and studies on the StudyU platform comply with ethical principles and international regulations, particularly the GDPR (European Union) 2016/679. Each participant is assigned an anonymous user account with a random ID assigned to the participant, eliminating the need for identifiable user profiles or app logins and enabling easy and user-friendly daily health parameters collection. This allows an individual and statistical analysis of the intervention’s effect, which is then reported back to participants to strengthen their confidence.

The patient’s consent must explicitly cover the collection and processing of personal data. Personal data and test results are protected. Participants are informed that their disease-related data and test results will be stored pseudonymously at the respective study centers and used for scientific evaluations. Data will not be shared with third, not actively involved parties. Data collected via StudyU will be stored on a server at HPI in anonymized form and published anonymously via the platform. All other data will be deleted after 10 years.

### Data Monitoring

The data monitoring committee consists of a medical doctor, a study nurse and a bioinformatics expert. The committee monitors the study visit schedules at both centers and the assessment including patient-reported outcomes. The 2 recruiting centers are required to report any changes to the study visit schedules or deviations from the study protocol to the monitoring committee. In the event of protocol deviations, the committee will report to the study coordinators. The Data Monitoring Committee is free from competing interests. Further details on the functioning of the committee can be obtained by emailing the corresponding author.

### Harms

Fasting should be discontinued immediately if serious adverse events occur (eg, neurological deficits as an indication of hyponatremia). In that case, patients will be given immediate medical care or referred to emergency care.

Each adverse event must be documented in detail if, in the opinion of the investigator, there is a causal relationship with the intervention. The documentation includes the type of event, severity, onset, duration, causality and changes for the study. Related signs and symptoms should be summarized as a single disease. The “adverse events study patient” form is available for documentation purposes. All adverse events must be tracked until they subside or stabilize.

Serious adverse events will be reported to the study coordinator and principal investigator as soon as they become known, that is, within 24 hours. The principal investigator, in close collaboration with the data monitoring committee and the study coordinator, may decide to discontinue the study, either due to adverse or serious adverse events attributable to the study intervention, or for the reasons mentioned in the section “exclusion criteria,” or because not enough patients could be recruited. Interim analyses are not conducted as no adverse health effects are expected.

## Discussion

This is the first study to investigate the feasibility of prolonged therapeutic fasting on AIMSS/AIA among breast cancer patients receiving aromatase inhibitor treatment. While the evidence for nutritional interventions in cancer care continues to grow, research specifically addressing fasting in the context of AIMSS/AIA remains sparse. To date, various nutritional interventions have demonstrated positive effects on treatment tolerance and symptom burden, particularly as complementary approaches to the conventional treatments for breast cancer patients. Based on this positive evidence, our study seeks to address the gap in clinical research in this unique context by exploring the feasibility and preliminary effects of fasting.

### Strengths

Fasting has been shown in other studies to be an effective, cost-efficient, and safe intervention for breast cancer patients and a way of introducing healthy habits.^61,63^

While our intervention specifically focuses on fasting (consisting of vegetable juices and broths, max. 350 kcal/day) many of the described molecular mechanisms have been extensively studied in the context of calorie restriction (CR). These mechanisms include the downregulation of proinflammatory cytokines,^64,65^ macrophage polarization toward an anti-inflammatory M2 state, and the promotion of autophagy.^66-68^ Emerging evidence suggests that similar physiological responses are activated during fasting.^69^ Moreover, fasting improves gut barrier integrity, reduces bacterial toxin absorption, and fosters protective bacteria in the microbiota.^70,71^ These systemic effects may contribute to reducing inflammation and enhancing overall wellbeing in breast cancer patients. Our study design incorporates validated fasting protocols supported by continuous counseling, ensuring qualified and accessible supervision. The short duration of the fasting intervention and the outpatient care with online meetings and the group environment are designed to maximize participant compliance through easy accessibility and peer support, laying the groundwork for potential translational implementation in everyday clinical practice. Nutritional counseling at *t*1 helps to address the common challenge of maintaining long-term behavioral changes.

One of the distinguishing features of this intervention is that it not only emphasizes fasting but also integrates important components such as mindfulness and exercise, thus offering a more holistic approach. Physical activity benefits patients by improving quality of life and mindfulness,^26^ reducing anxiety, depression, and fatigue, and enhancing cardiorespiratory fitness and muscular strength.^72,73^ Exercising while fasting can optimize glucose regulation, enhance lipid oxidation, and reduce systemic inflammation, collectively improving key health outcomes. The body draws on stored glycogen, fats and proteins as energy sources^74^ and switches toward fat oxidation and lipolysis, supporting energy needs through gluconeogenesis and ketogenesis.^74-76^ Previous studies have also described positive effects on the gut microbiota, including bowel motility and intestinal blood flow. By strengthening the intestinal barrier, the mucosal immune cells help to reduce low-grade inflammation and maintain overall gut integrity.^77^ Furthermore, in fibromyalgia patients, low-speed exercises minimize the impact on joints, enhance microcirculation and promote the elimination of oxygen free radicals.^78^ By reducing peripheral nociceptive input, they disrupt the pain cycle, leading to a more effective pain management.^78^

Additionally, this study captures a comprehensive range of exploratory outcomes, including anthropometric data and patient-related outcomes such as quality of life and fatigue. These measurements, complemented by electronic data monitoring via the StudyU app, enable the collection of complete datasets and provide feedback to participants on their individual progress, motivating them to hold on to lifestyle changes. The flexibility and user-friendly design of the StudyU app enhance participant engagement while adhering to rigorous data protection standards.

By incorporating validated tools such as scales, questionnaires and blood biomarkers, our study creates a basis for identifying potential outcome measures for future studies. BIA measurements allow a better insight into potential effects of fasting on body composition.

Observing feasibility in this pilot study will lay the groundwork for a confirmatory follow-up study, including a control group and adjusted endpoints, to provide more robust and conclusive evidence.

### Limitations

This study acknowledges some limitations. As this is an exploratory pilot design that lacks preliminary studies, we deliberately decided not to randomize participants to first determine whether the intervention is feasible at all. However, incorporating a control group in future studies could provide a baseline for comparison, enhancing the validity and reliability of the findings. This would also allow robust statistical analyses and confidence in the findings.

Blinding, while inherently challenging in fasting interventions due to their nature, remains a potential limitation as it could influence participant behavior and subjective outcomes.

Another limitation is the lack of a systematic record of participants’ exercise habits and the use of liver wraps or fasting aids. Additionally, we cannot objectively confirm their transition into fasting metabolism through glucose or ketone monitoring. Such markers should be included in a confirmatory study to gain a better understanding of the effects of fasting over time. Furthermore, not precisely tracking post-fasting dietary intake is a drawback, as this data would be crucial for assessing the potential duration of fasting effects.

Adherence to the fasting protocol in an outpatient setting is another challenge as it relies on self-reporting, which is prone to inaccuracies. Additionally, participants’ awareness of the study objectives and the generally favorable perception of fasting in Germany could introduce bias, potentially influencing both behavior and outcomes. Furthermore, the short follow-up period limits the assessment of long-term effects of fasting interventions. Lastly, the lack of additional objective measures, such as continuous glucose monitoring or inflammatory biomarker profiling, restricts the physiological insights that can be drawn from this intervention.

## Conclusion

In conclusion, our planned intervention highlights both the need for and potential of dietary interventions as cost-effective, safe and supportive elements of an integrative therapeutic approach for breast cancer patients and contributes to a better understanding of the pathogenetic mechanisms of treatment-related side effects.

To draw more definitive conclusions, further clinical trials with larger sample sizes, randomization and control groups will be needed to investigate the long-term effects of fasting on symptom control in AIMSS/AIA, as well as other endpoints such as fatigue and sleep.

## Supplemental Material

sj-docx-1-ict-10.1177_15347354261426272 – Supplemental material for Therapeutic Fasting as a Novel Approach to Mitigate Musculoskeletal Symptoms in Breast Cancer Patients undergoing Aromatase Inhibitor Therapy: A Feasibility Study Protocol Supplemental material, sj-docx-1-ict-10.1177_15347354261426272 for Therapeutic Fasting as a Novel Approach to Mitigate Musculoskeletal Symptoms in Breast Cancer Patients undergoing Aromatase Inhibitor Therapy: A Feasibility Study Protocol by Henriette Meyer, Daniela A. Koppold, Osaid Shaker, Laura Eden, Lisa Schiffmann, Stefan Konigorski, Holger Cramer, Kyung-Eun Anna Choi, Hermann Einsele, Marcela Winkler and Claudia Löffler in Integrative Cancer Therapies

sj-docx-2-ict-10.1177_15347354261426272 – Supplemental material for Therapeutic Fasting as a Novel Approach to Mitigate Musculoskeletal Symptoms in Breast Cancer Patients undergoing Aromatase Inhibitor Therapy: A Feasibility Study Protocol Supplemental material, sj-docx-2-ict-10.1177_15347354261426272 for Therapeutic Fasting as a Novel Approach to Mitigate Musculoskeletal Symptoms in Breast Cancer Patients undergoing Aromatase Inhibitor Therapy: A Feasibility Study Protocol by Henriette Meyer, Daniela A. Koppold, Osaid Shaker, Laura Eden, Lisa Schiffmann, Stefan Konigorski, Holger Cramer, Kyung-Eun Anna Choi, Hermann Einsele, Marcela Winkler and Claudia Löffler in Integrative Cancer Therapies

sj-docx-3-ict-10.1177_15347354261426272 – Supplemental material for Therapeutic Fasting as a Novel Approach to Mitigate Musculoskeletal Symptoms in Breast Cancer Patients undergoing Aromatase Inhibitor Therapy: A Feasibility Study Protocol Supplemental material, sj-docx-3-ict-10.1177_15347354261426272 for Therapeutic Fasting as a Novel Approach to Mitigate Musculoskeletal Symptoms in Breast Cancer Patients undergoing Aromatase Inhibitor Therapy: A Feasibility Study Protocol by Henriette Meyer, Daniela A. Koppold, Osaid Shaker, Laura Eden, Lisa Schiffmann, Stefan Konigorski, Holger Cramer, Kyung-Eun Anna Choi, Hermann Einsele, Marcela Winkler and Claudia Löffler in Integrative Cancer Therapies

sj-docx-4-ict-10.1177_15347354261426272 – Supplemental material for Therapeutic Fasting as a Novel Approach to Mitigate Musculoskeletal Symptoms in Breast Cancer Patients undergoing Aromatase Inhibitor Therapy: A Feasibility Study Protocol Supplemental material, sj-docx-4-ict-10.1177_15347354261426272 for Therapeutic Fasting as a Novel Approach to Mitigate Musculoskeletal Symptoms in Breast Cancer Patients undergoing Aromatase Inhibitor Therapy: A Feasibility Study Protocol by Henriette Meyer, Daniela A. Koppold, Osaid Shaker, Laura Eden, Lisa Schiffmann, Stefan Konigorski, Holger Cramer, Kyung-Eun Anna Choi, Hermann Einsele, Marcela Winkler and Claudia Löffler in Integrative Cancer Therapies

sj-docx-5-ict-10.1177_15347354261426272 – Supplemental material for Therapeutic Fasting as a Novel Approach to Mitigate Musculoskeletal Symptoms in Breast Cancer Patients undergoing Aromatase Inhibitor Therapy: A Feasibility Study Protocol Supplemental material, sj-docx-5-ict-10.1177_15347354261426272 for Therapeutic Fasting as a Novel Approach to Mitigate Musculoskeletal Symptoms in Breast Cancer Patients undergoing Aromatase Inhibitor Therapy: A Feasibility Study Protocol by Henriette Meyer, Daniela A. Koppold, Osaid Shaker, Laura Eden, Lisa Schiffmann, Stefan Konigorski, Holger Cramer, Kyung-Eun Anna Choi, Hermann Einsele, Marcela Winkler and Claudia Löffler in Integrative Cancer Therapies
