# Supplementary material for: Therapeutic Fasting as a Novel Approach to Mitigate Musculoskeletal Symptoms in Breast Cancer Patients undergoing Aromatase Inhibitor Therapy: A Feasibility Study Protocol
Source: Integr Cancer Ther. 2026 Mar 10;25:15347354261426272. doi: 10.1177/15347354261426272 (PMC12979920; doi:10.1177/15347354261426272)
Supplement: sj-docx-1-ict-10.1177_15347354261426272 – Supplemental material for Therapeutic Fasting as a Novel Approach to Mitigate Musculoskeletal Symptoms in Breast Cancer Patients undergoing Aromatase Inhibitor Therapy: A Feasibility Study Protocol [file sj-docx-1-ict-10.1177_15347354261426272.docx]

Declaration of consent for participation in the scientific study on

**Explorative study on therapeutic fasting to reduce limitations on physical well-being and quality of life under endocrine therapy with aromatase inhibitors (FREE-AI)**

_________________________________ ______________________

Surname, First name Date of birth

Your personal data and medical findings will be collected for this scientific project. The transfer, storage and evaluation of this study-related data is pseudonymized and in accordance with the legal provisions of the European General Data Protection Regulation (GDPR) and requires the following voluntary consent before participation:

I hereby declare that I have been informed both orally and in written form about the content, objectives, procedure, significance and effects of the above-mentioned study by _______________________. I had sufficient opportunity to ask questions and my questions about this study were answered to my satisfaction during the discussion with the study doctor. I had sufficient time to freely decide whether or not to participate in this study.

I am aware that participation in the study is voluntary and that I can withdraw my consent at any time without giving reasons and without incurring any disadvantages. In this case, I will contact the study doctor and inform them of my decision. If the data has not yet been analyzed and/or published, the data collected from me will then be automatically deleted from the study data. It is not possible to delete data that has already been analyzed and/or published.

I agree that data/disease data collected from me as part of this study may be stored in pseudonymized form and scientifically evaluated in pseudonymized form. The confidentiality of the collected and stored data of the study participant is also guaranteed in the event of publication. The storage period of the data is 10 years.

Privacy policy of the study

I am aware that my personal data, in particular medical findings, will be evaluated during the planned study.

1. I consent to the data collected in the course of this clinical study, in particular information about my health, being recorded, scientifically recorded and evaluated in paper form and on electronic data carriers by the study physician at the University Hospital of Würzburg. If necessary, the collected data may be pseudonymized and passed on to non-employees of the clinic for the purpose of scientific evaluation.
2. I agree that my data will be stored for 10 years after the end of the study. After that or after withdrawal of my consent to participate in the study, my personal data will be deleted, unless there are legal or statutory retention periods to the contrary.
3. Furthermore, I authorize the study staff to contact me or my relatives in writing or by telephone for a telephone follow-up interview and to make an appointment for the personal follow-up examinations and follow-up interviews at the University Hospital of Würzburg.

I agree that the study results will be published in anonymized form so that no conclusions can be drawn about my identity.

I have been given a copy of the data protection and patient information and this declaration of consent.

______________________ __________________________________________________

Place, Date Patient’s signature

Declaration of the study doctor or project staff member

I, _________________________, hereby declare to have informed the above-mentioned patient on _ _. _ _ . _ _ _ _ _ _ about the nature, significance, consequences, risks and participation rights in the study “Therapeutic fasting to reduce restrictions on physical well-being and quality of life under endocrine therapy with aromatase inhibitors (FREE-AI)” orally and in written form and to have given them a copy of the patient information and this declaration of consent.

______________________ __________________________________________________

Place, Date Signature of the doctor informing the patient
