# Supplementary material for: Therapeutic Fasting as a Novel Approach to Mitigate Musculoskeletal Symptoms in Breast Cancer Patients undergoing Aromatase Inhibitor Therapy: A Feasibility Study Protocol
Source: Integr Cancer Ther. 2026 Mar 10;25:15347354261426272. doi: 10.1177/15347354261426272 (PMC12979920; doi:10.1177/15347354261426272)
Supplement: sj-docx-2-ict-10.1177_15347354261426272 – Supplemental material for Therapeutic Fasting as a Novel Approach to Mitigate Musculoskeletal Symptoms in Breast Cancer Patients undergoing Aromatase Inhibitor Therapy: A Feasibility Study Protocol [file sj-docx-2-ict-10.1177_15347354261426272.docx]

**Handling of ongoing treatments and adapting medication**

If the patient is on medication while participating in the fasting intervention, certain medications may need to be adjusted accordingly.

1. **Oral antidiabetics**

All oral antidiabetic medications – including biguanides (metformin), sulfonylureas, glinides, glitazones, α-glucosidase inhibitors, DPP-4 inhibitors and SGLT-2 inhibitors – should be discontinued from the beginning of the fasting until it ends.

Patients should check their blood glucose levels daily for the first three days of fasting. If levels exceed 12 mmol/L (216 mg/dL), they should seek medical advice and advise the study director.

It is important to emphasize that temporary blood sugar spikes up to 12 mmol/L are not dangerous. However, taking these medications while fasting can lead to hypoglycemia, which is potentially life-threatening.

1. **Anticoagulants**

- **Marcumar:** Reduce the dose by half on the third day of fasting, then resume the regular dose.
- **NOACs:** Continue taking as usual; no adjustments are needed while fasting.

1. **Antihypertensives**

- **ACE inhibitors and Sartans:** Reduce the dose by half on fasting days. If the dose is already minimal, discontinue temporarily on fasting days.
- **Diuretics:** Please eliminate these as they can disturb the fluid and electrolyte balance.
- **Combination Medications (containing diuretics)**: Prescribe the other components separately at half the usual dose on fasting days as the diuretics should be discontinued.
- **Beta-blockers:** Do not discontinue!

Patients who own a blood pressure monitor should be encouraged to track their blood pressure at home regularly.

1. **Migraine management during fasting**

Patients prone to migraines should be aware that fasting may trigger attacks. Therefore, it is important that they take their usual preventative medication (e.g. triptans) as soon as the first signs of a migraine appear.
