# Supplementary material for: Therapeutic Fasting as a Novel Approach to Mitigate Musculoskeletal Symptoms in Breast Cancer Patients undergoing Aromatase Inhibitor Therapy: A Feasibility Study Protocol
Source: Integr Cancer Ther. 2026 Mar 10;25:15347354261426272. doi: 10.1177/15347354261426272 (PMC12979920; doi:10.1177/15347354261426272)
Supplement: sj-docx-3-ict-10.1177_15347354261426272 – Supplemental material for Therapeutic Fasting as a Novel Approach to Mitigate Musculoskeletal Symptoms in Breast Cancer Patients undergoing Aromatase Inhibitor Therapy: A Feasibility Study Protocol [file sj-docx-3-ict-10.1177_15347354261426272.docx]

**Dear participant,**

**Welcome to the FREE-AI study!**

On the following pages you will find information, recipe suggestions and tips to help you prepare as well as possible for the fasting intervention.

During the two preparatory days, you should eat easily digestible, high-fiber foods and slowly reduce the amount of food to gently prepare you and your digestive tract for the following food deprivation. Stimulants like coffee, black/green tea, nicotine, sweets and alcohol that are not consumed during the fasting week should also be avoided. If possible, reduce your coffee intake cup by cup before the first day of preparation. This will make it easier for you to start the fasting week and prevent unpleasant side effects such as headaches, nausea and circulatory problems.

During the first few days of fasting, it is a good idea to avoid potentially stressful activities, e.g. shopping. We encourage you to buy the food in advance.

| **Day 1 – Day 2** | **Day 3 – Day 9** | **Day 10 – Day 12** |
| --- | --- | --- |
| Preparatory Days | Fasting days | Recovery days |
| Slowly decrease your food intake.  Avoid stimulants.  Focus on high-fiber, easily digestible carbohydrates. | Consume vegetable juice and vegetable broths.  Drink tea and water. | Slowly increase your food intake.  Include carbohydrates,  keep protein and fat low. |

1. Preparatory days (Day 1-2)

It should be noted that cooked meals are often tolerated better than raw food.

**Rice day**

Cook 50 g whole grain basmati rice or 50 g millet or 50 g quinoa in 100 ml water three times a day. Serve in the morning with 200 g of berries or 150 g of freshly grated, unpeeled apple or at lunchtime and in the evening with 200 g of stewed tomatoes or other vegetables.

Season with herbs (fresh parsley or basil).

**Potato day**

600 - 700 g potatoes divided between three meals. The potatoes should be prepared as jacket or baked potatoes, for example with fresh herbs (marjoram, parsley, thyme, chives, dill or caraway). Each meal should be accompanied by approx. 200 g of vegetables, e.g. freshly sliced tomatoes with chives, lemon and curry in the morning, steamed vegetables and fresh herbs at lunchtime and in the evening.

**Vegetable day**

Breakfast: Mix 200 g grated carrots and 1 medium-sized grated apple and season with lemon juice, cinnamon or pure vanilla powder. (Refine with 1 tsp olive or linseed oil if necessary).

Lunch e.g.: 200 g steamed cauliflower and 100 g steamed broccoli or 200 g steamed pumpkin and 100 g steamed kohlrabi, with 1 medium-sized potato.

Evening e.g.: 50 g salad (e.g. chicory, iceberg, lamb's lettuce), 1 medium-sized tomato, 6 radishes, 1⁄4 green cucumber seasoned with lemon juice, fresh herbs, pepper, 1 tsp olive or linseed oil if necessary.

**Oat day**

Three times a day, briefly soak 50 g wholegrain oat flakes in a little water with 200 g berries or 150 g freshly grated, unpeeled apple, seasoned with cinnamon and pure vanilla powder. Alternatively, serve with 200 g each of steamed vegetables and fresh herbs.

This is an example of what a preparatory day could look like:

**Day A**

Breakfast:

A cup of herbal tea or fasting tea, a small bowl of porridge

(prepared with water or oat milk) with berries.

Lunch:

A medium-sized potato with easily digestible steamed vegetables (e.g. carrot, zucchini, tomato, pumpkin, parsnip).

Dinner:

200g steamed cauliflower and 100g steamed broccoli with herbs or 1 plate of vegetable soup.

**Day B**

Breakfast:

A cup of herbal tea or fasting tea, a small bowl of porridge

(prepared with water or oat milk) with berries or apple.

Lunch:

Brown rice with easily digestible steamed vegetables or 50 g salad (e.g. chicory, iceberg lettuce, lamb's lettuce), 1 medium-sized tomato, 6 radishes, 1⁄4 green cucumber seasoned with lemon juice, fresh herbs, white pepper and a little sea salt, if necessary, with 1 tsp olive or linseed oil.

Dinner:

A bowl of cold or warm vegetable soup with easily digestible vegetables, pureed into cream soup.

1. Recipes for the fasting days (Day 3-9)

**In the morning:** 150 ml vegetable juice (e.g. carrot juice, beet juice)

**At lunchtime:** 150 ml vegetable juice (e.g. tomato juice)

**In the evening:** approx. 250 ml of home-made broth

The juice and broth can also be alternated.

Drink 2 - 3 liters of water and herbal tea throughout the day, you can also squeeze a lemon and drink it or bite on lemon slices

Tips & tricks:

- The broth can also be replaced by tomato juice diluted with hot water. Pay attention to the salt content. The juice can be spooned carefully and consumed warm, if necessary.
- Add variety to your meals by using different types of vegetables.
- If you want a little more flavor in your water, you can add cucumber slices, lemon slices or mint leaves (“infused water”).
- If your stomach is sensitive or you tend to feel cold, you can also take warm oat or linseed gruel instead of juice. To do this, add 1 - 2 tablespoons of fine oat flakes or, if possible, light-colored, ground linseed to 250 ml water. Boil for 5 minutes or leave to stand for longer in the hot boiled water and stir. Before drinking, strain out the solid parts!

Some suggestions for fresh, home-pressed juices:

- Carrot - lemon - ginger
- Cucumber - celery - mint
- Beetroot – fennel

Vegetable broths

Recipes are each for 4 portions

**Basic vegetable stock recipe**

1 liter of water

1 kg vegetables (e.g. carrots, tomatoes, pumpkin, broccoli, zucchini)

1 clove

3 -4 bay leaves

4 -5 juniper berries

1 -2. tbsp fresh herbs (e.g. parsley, oregano, marjoram, basil, dill, lovage, sea salt if necessary)

Wash and chop the vegetables, bring the water to the boil and add the vegetables. Cook the stock over a low heat for 60 - 90 minutes. After 15 minutes of cooking, add bay leaves, clove and juniper berries and leave to simmer. Strain, season to taste with fresh herbs and a pinch of sea salt if necessary.

TIP

Add fresh tomatoes to the stock and season with 1 - 2 teaspoons of tomato paste and fresh basil.

**Potato stock**

1 liter of water

250 g potatoes

250 g carrots

1 stick of leek

1 large parsnip

3 -4 bay leaves

1⁄2 tsp caraway seeds

4-5 juniper berries

parsley and sea salt (approx. 1 tsp)

Wash and chop vegetables, bring the water to the boil and add the vegetables. Cook on the lowest heat for 60 - 90 minutes. After 15 minutes of cooking, add the caraway seeds and leave the soup to simmer. Strain and season to taste with finely chopped parsley and a pinch of sea salt. Other seasoning options are dill, basil, lovage, nutmeg, marjoram or oregano.

Liver compress

A midday rest with a liver compress can help you get through a day of fasting more easily.

For this you need:

- 1 - 2 heat carriers, e.g. grain pillows
- 1 tea towel
- 1 bath towel
- 1 bowl that fits a tea towel
- 1 blanket

How it works:

- Prepare 2 grain pillows or an alternative heat carrier. Heat in the microwave or oven according to the instructions.
- Dip a tea towel into the bowl of hot water and wring it out well.
- Place the double or triple folded towel on the right upper abdomen under the ribcage and the heat carriers over it. Secure with a bath towel or a tight T-shirt.
- Lie down for 30 minutes and enjoy. Cover yourself well so that you are comfortably warm.
- If necessary, you can place a second heat transfer medium under your feet.

Exercise and relaxation

During your fasting week, sufficient exercise in the fresh air as well as periods of rest are important to support the optimal fasting process.

Exercise promotes fat burning, good circulation and breathing activity. You can prevent muscle loss by doing gymnastic exercises and going for a walk, cycling or swimming. If you are used to other types of sport, you can also do these carefully during fasting. If possible, exercise for at least one hour a day.

At the same time, regular rest periods are just as important. In addition to the rest phase during the liver wrap, other relaxation exercises such as autogenic training, light yoga, Qi Gong exercises or meditation are ideal. If possible, make sure you are breathing deeply.

1. Recovery days (Day 10 – Day 12)

The recovery days are important, but also more difficult than fasting - therefore special attention should be paid to these days. The transition should be slow, gentle and gradual. The digestive tract must first gradually get used to eating again.

Please eat small portions, chew your food thoroughly and enjoy it consciously. During the recovery days, use as many herbs as possible and very little salt.

To stimulate the intestines again, eat lots of fiber. A cup of swollen, preferably light-colored linseed is also recommended in the morning. Soak 1 tablespoon of linseed (alternatively: oat flakes) in 250 ml overnight or briefly boil 1 tablespoon in 250 ml of water and allow it to swell well.

Make sure you continue to drink enough, exercise lightly and relax.

**Day 10:** **only food high in carbohydrates**

Morning: 1 apple, optionally soaked oats or flaxseed

Lunch: vegetable soup

Dinner: steamed carrot with herbs, optionally crisp bread

**Day 11:** **Carbohydrate-rich foods, little protein**

Morning: porridge with fresh fruits

Lunch: potatoes or millet with vegetables and herbs

Dinner: vegetable-potato soup with pumpkin seeds OR crisp bread with vegan spread and vegetables

**Day 12: Carbohydrate-rich foods, little protein, little fat**

Morning: porridge with fresh fruits

Lunch: raw vegetable salad OR rice and vegetables, 1 tablespoon of oil

Dinner: crispbread/wholegrain bread with natural tofu or plant-based cream cheese or vegan spread with vegetables; alternatively: potato soup
