# Supplementary material for: Therapeutic Fasting as a Novel Approach to Mitigate Musculoskeletal Symptoms in Breast Cancer Patients undergoing Aromatase Inhibitor Therapy: A Feasibility Study Protocol
Source: Integr Cancer Ther. 2026 Mar 10;25:15347354261426272. doi: 10.1177/15347354261426272 (PMC12979920; doi:10.1177/15347354261426272)
Supplement: sj-docx-4-ict-10.1177_15347354261426272 – Supplemental material for Therapeutic Fasting as a Novel Approach to Mitigate Musculoskeletal Symptoms in Breast Cancer Patients undergoing Aromatase Inhibitor Therapy: A Feasibility Study Protocol [file sj-docx-4-ict-10.1177_15347354261426272.docx]

Welcome to your first questionnaire!

We ask you to fill in the following questions carefully and honestly. Your answers will have no influence on your participation in the study. If you have any questions, please contact your study doctor.

Thank you very much for your participation!

This survey contains 27 questions.

**General questions about yourself**

1. **Please enter your study ID: ***

Please enter your answer here:

**2. In which year were you born? ***

Only numbers may be entered in this field.

Please enter your answer here:

**Questions about fasting and exercise**

**3. Do you already have experience with fasting? ***

Please select one of the following answers:

Please select only one of the following answers:

- yes, once
- yes, several times
- no

**4. What type of fasting have you already practiced? ***

Only answer this question if the following conditions are fulfilled:

Answer was 'yes, several times' *or* 'yes, once' for question '[fasting experience]' (Have you already had fasting experience?)

Please choose one of the following answers:

Please select only one of the following answers:

- Juice fasting
- Fruit fasting
- Whey fasting
- Tea fasting
- Zero diet
- Other (enter in comment field)
- No entry

Please write a comment about your selection.

**5. How often have you fasted? ***

Only answer this question if the following conditions are fulfilled:

Answer was 'yes, several times' for question '[fasting experience]' (Have you already had experience of fasting?)

Please choose one of the following answers:

Please select only one of the following answers:

- less than 1 time a year
- 1-2 times a year
- 3-5 times a year
- 6-9 times a year
- more than 10 times a year

**6. How many days in a row have you fasted on average?**

Only answer this question if the following conditions are met:

Answer was 'yes, several times' for question '[fasting experience]' (Have you already had fasting experience?)

Only numbers may be entered in this field.

Please enter your answer here:

**7. What was reason for fasting? ***

Only answer this question if the following conditions are met:

Answer was 'yes, several times' or 'yes, once' for question '[fasting experience]' (Have you already had experience of fasting?)

Please choose one of the following answers:

Please select only one of the following answers:

- For health reasons
- For religious reasons
- For other reasons (please enter in the comment field)

Please write a comment about your selection

**8. Do you do sport or physically strenuous work (e.g. gardening)? ***

Please select only one of the following answers:

- Yes
- No

**9. How often do you exercise? ***

Only answer this question if the following conditions are met:

Answer was 'Yes' to question '[Sport]' (Do you do sport or physically strenuous work (e.g. gardening)?

Please select one of the following answers:

Please select only one of the following answers:

- Less than 1 time per week
- 1 time per week
- 2 to 3 times per week
- 4 to 6 times per week
- Daily

**10. How long do you exercise per unit? ***

Only answer this question if the following conditions are met:

Answer was 'Yes' to question '[Sport]' (Do you do sport or physically strenuous work (e.g. gardening)?

Please select one of the following answers:

Please select only one of the following answers:

- less than 30 minutes
- 30 to 60 minutes
- 60 to 90 minutes
- more than 90 minutes

**11. What is the estimated total duration of your sporting activity per week in minutes? ***

Only answer this question if the following conditions are met:

Answer was 'Yes' to question '[Sport]' (Do you do sport or physically strenuous work (e.g. gardening)?

Only numbers may be entered in this field.

Please enter your answer here:

**Questions about stimulants and eating habits**

**12. Do you smoke cigarettes? If yes, how many? ***

Please select one of the following answers:

Please select only one of the following answers:

- none
- less than 10 cigarettes per day
- less than 20 cigarettes per day
- more than 20 cigarettes per day

**13. What is your current diet? ***

Please select one of the following answers:

Please select only one of the following answers:

- With meat and fish
- With meat without fish
- Without meat with fish
- Vegetarian
- Vegan
- Other (enter in the comment field)

Please write a comment about your choice

**14. How much fruit and vegetables do you usually eat per day? (1 portion corresponds to approx. 100g/ 5 portions = 500g per day) ***

Please choose one of the following answers:

Please select only one of the following answers:

- 5 portions or more per day (100%)
- 3-4 portions daily (75%)
- 2-2.5 portions daily (50%)
- 1-1.5 portions daily (25%)
- rarely or not at all

**15. How often do you use whole grain products (bread, rice, pasta)? ***

Please select one of the following answers:

Please select only one of the following answers:

- exclusively (100%)
- predominantly (75%)
- about half (50%)
- occasionally (25%)
- rarely or not at all

**16. How often do you usually eat cold meats? ***

Please select one of the following answers:

Please select only one of the following answers:

- 2-3 times a day
- about once a day
- not daily (3-5 times per week)
- occasionally (1-2 times a week)
- rarely or not at all

**17. How often do you usually eat meat? ***

Please select one of the following answers:

Please select only one of the following answers:

- 2-3 times a day
- about once a day
- not daily (3-5 times per week)
- occasionally (1-2 times per week)
- rarely or not at all

**18. How often do you usually eat cheese? ***

Please select one of the following answers:

Please select only one of the following answers:

- 2-3 times a day
- about once a day
- not daily (3-5 times per week)
- occasionally (1-2 times a week)
- rarely or not at all

1**9. How often do you usually eat confectionery (cakes, ice cream, chocolate ...)? ***

Please choose one of the following answers:

Please select only one of the following answers:

- (almost) never
- approx. 1-3 times a month
- approx. 1 time per week
- approx. every other day
- every day

**20. How often do you usually eat fast food or convenience foods (chips, hamburgers, pizza, sausages, cold meats, canned or tinned food)? ***

Please choose one of the following answers:

Please select only one of the following answers:

- (almost) never
- approx. 1-3 times a month
- approx. 1 time per week
- approx. every other day
- daily

**21. How often do you usually eat nuts? ***

Please choose one of the following answers:

Please select only one of the following answers:

- (almost) never
- approx. 1-3 times a month
- approx. 1 time per week
- approx. every other day
- daily

**22. How often do you use high-quality oils with a high omega-3 fatty acid content (flaxseed oil, hemp oil, walnut oil, rapeseed oil, soybean oil)? ***

Please select one of the following answers:

Please select only one of the following answers:

- exclusively 100%
- predominantly 75%
- about half 50%
- occasionally 25%
- rarely or not at all

**23. To what extent does the following statement apply to you: I usually eat very quickly. ***

Please select one of the following answers:

Please select only one of the following answers:

- strongly agree
- rather true
- partly/partly
- rather not applicable
- does not apply at all

**24. How often do you drink coffee? ***

Please select one of the following answers:

Please select only one of the following answers:

- (almost) never
- approx. 1-3 times a month
- approx. 1 time per week
- approx. every other day
- daily

**25. How often do you drink soft drinks (cola, lemonade, Fanta, soda ...)? ***

Please choose one of the following answers:

Please select only one of the following answers:

- (almost) never
- approx. 1-3 times a month
- approx. 1 time per week
- approx. every other day
- daily

**26. How often do you drink alcoholic beverages? ***

Please select one of the following answers:

Please select only one of the following answers:

- (almost) never
- approx. 1-3 times a month
- approx. 1 time per week
- approx. every other day
- daily

**27. How often do you drink herbal teas or water? ***

Please select one of the following answers:

Please select only one of the following answers:

- (almost) never
- approx. 1-3 times a month
- approx. 1 time per week
- approx. every other day
- daily

Thank you, you have now successfully completed the survey!

Thank you for answering the questionnaire.

The same questions will be asked again at t2 (3 months after t0). Questions on fasting (3-7) will not be asked at t2.
