# Supplementary material for: Therapeutic Fasting as a Novel Approach to Mitigate Musculoskeletal Symptoms in Breast Cancer Patients undergoing Aromatase Inhibitor Therapy: A Feasibility Study Protocol
Source: Integr Cancer Ther. 2026 Mar 10;25:15347354261426272. doi: 10.1177/15347354261426272 (PMC12979920; doi:10.1177/15347354261426272)
Supplement: sj-docx-5-ict-10.1177_15347354261426272 – Supplemental material for Therapeutic Fasting as a Novel Approach to Mitigate Musculoskeletal Symptoms in Breast Cancer Patients undergoing Aromatase Inhibitor Therapy: A Feasibility Study Protocol [file sj-docx-5-ict-10.1177_15347354261426272.docx]

**Focus group guidelines**

for participation in the

**"Exploratory study on therapeutic fasting to reduce physical limitations and quality of life under endocrine therapy with aromatase inhibitors" (FREE-AI)**

| **A: Procedure and introduction round** |
| --- |
| *Welcome to the focus group for participants in the FREE-AI study. We have come together today in this digital space to create a platform for exchange where you can share your personal experiences. Looking back, how did you find the fasting experience? How did participating in the study affect you? We are also interested in continuously improving. For example, we want to learn from you what we as a research team could have done differently in the course of the study. At this point, it is important to me to emphasize that there are no "right" or "wrong" answers in the course of our discussion. Your personal experiences will be the focus of our interest in the next 60 to 90 minutes.*  **Introductions:** Let's briefly introduce ourselves. Please briefly mention when you completed the 7-day fasting intervention and why you decided to participate in today's group discussion.  **Icebreaker:** Please complete the following sentence: "If I had to summarize the meaning of therapeutic fasting for me personally in 3 words, they would be X, Y, Z."   - In-depth discussion: Reference to the words mentioned, topics, and further exploration |
| **B.1 Experience of fasting** |
| **How did you personally experience the week of fasting that you completed as part of your participation in the FREE-AI study? How did you feel during this time?**   - To what extent did you feel influenced by fasting? - How did fasting affect your everyday life? - Were there moments when you found fasting difficult? If so, what helped you in such situations?   **What role did people in your personal and professional environment play during the fasting week?**   - To what extent were you supported by those around you during the fast? What else would you have liked to have received from those around you? - Did you feel you had to explain yourself to those around you? If so, how did you deal with this? - How did you deal with social situations in which food plays a decisive role (e.g., restaurant visits with friends, birthdays)? |
| **B.2 Study procedure** |
| **Next, we are interested in how you became aware of the Free-AI study. How did you come to participate in the study?**  **How did you find the study process?**   - Looking back, how would you rate your personal contact with the study team? - How well did you feel you were treated and cared for before, during, and after fasting? - Did you feel sufficiently informed? Would you have liked more information?   **You answered questions about your general well-being every day in the Study-U app. How would you rate the user-friendliness of the app?**   - Did you encounter any problems installing, using, or operating the app on a daily basis? Were you able to integrate its use into your everyday life? - Which elements of the app did you find particularly helpful? - Do you have any suggestions for changes that would make the app more user-friendly for study purposes? Are there any alternatives you would like to see here?   **Now that some time has passed since you fasted, how would you personally rate the fasting experience?**   - Have you fasted again on your own initiative in the meantime, or to what extent do you plan to do so? - To what extent have your eating habits changed since fasting (in the long term)? - To what extent have there been other changes that you attribute to fasting? - To what extent would you advise other patients to fast or advise them against it? |
| **B.3 Praise and criticism** |
| **What aspects of participating in the study did you particularly like?**  **What would you like to see if you could participate in this study again?**   - Do you have any wishes regarding the care provided by the study team? - Would you have liked to receive further information? |
| **Conclusion** |
| **We have come to the end of our conversation. I would like to thank you very much for your openness and participation. Finally, are there any other experiences or wishes that you would like to share in connection with your participation in the study that have not yet been addressed?**  **Thank you very much!** |
